# Age and diabetic complications in type 2 diabetes: the role of disease duration and stage-dependent effects

**DOI:** 10.3389/fendo.2026.1782406

**Published:** 2026-05-08

**Authors:** Xu Zhang, Hongbin Song, Mingzhong Ji, Xiaojuan Luan, Jingzhu Nan, Yanhong Gao

**Affiliations:** 1 Department of Clinical Laboratory, the 1st Medical Center, Chinese PLA General Hospital, Beijing, China; 2 Department of Clinical Laboratory, the 3rd Medical Center, Chinese PLA General Hospital, Beijing, China; 3 Chinese PLA Medical School, Beijing, China; 4 Department of Clinical Laboratory, Qingdao Third People’s Hospital, Shandong, Qingdao, China; 5 Department of Clinical Laboratory, Beijing Anzhen hospital, Capital Medical University, Beijing, China

**Keywords:** Age, diabetic complications, disease duration, interaction, microvascular disease, restricted cubic spline, type 2 diabetes mellitus

## Abstract

**Background:**

Diabetic complications represent a major source of morbidity and mortality in patients with type 2 diabetes mellitus (T2DM). However, the role of age in the development and progression of complications across different stages remains incompletely understood.

**Methods:**

This cross-sectional study included 489 patients with T2DM. Diabetic complications were classified as macrovascular disease (MaVD), microvascular disease (MVD), and peripheral neuropathy (PN). The number of complications (0, 1, 2, ≥3) was used to assess complication burden. Logistic regression, partial proportional odds models, interaction analysis, and restricted cubic spline (RCS) models were applied to evaluate the association between age and diabetic complications.

**Results:**

The overall prevalence of complications was 75.66% and increased progressively with age. Age was independently associated with the presence of diabetic complications after adjustment for confounders (OR = 1.06, 95% CI: 1.03–1.08). The effect of age was more pronounced during the transition from no complications to multiple complications, but attenuated at higher stages. Subtype analysis showed that age was most consistently associated with microvascular disease, while the association with peripheral neuropathy was borderline and not significant for macrovascular disease after full adjustment. A significant interaction between age and diabetes duration was observed, indicating that the effect of age was stronger in early-stage disease but diminished with longer duration. RCS analysis demonstrated a positive and approximately linear association between age and complication risk.

**Conclusions:**

Age is independently associated with diabetic complications in T2DM, particularly in the early and intermediate stages of complication development. The effect of age varies by complication subtype and is modified by diabetes duration.

## 1 Introduction

Type 2 diabetes mellitus (T2DM) is a prevalent and progressive metabolic disorder characterized by chronic hyperglycemia due to impaired insulin secretion, insulin resistance, or both (1, 2). The global burden of T2DM has increased substantially over recent decades, particularly in developing countries such as China, where the prevalence has exceeded 10% in adults (3). Moreover, the age of onset is gradually decreasing, with an increasing number of younger individuals being affected (4).

Persistent hyperglycemia leads to widespread damage in both macrovascular and microvascular systems, as well as the peripheral nervous system (5). As the disease progresses, patients face an increased risk of developing a broad spectrum of complications, including cardiovascular disease, diabetic nephropathy, retinopathy, and peripheral neuropathy (6, 7). These complications often accumulate over time, resulting in a progressively increasing burden of disease that significantly impairs quality of life and increases mortality.

While previous studies have identified multiple risk factors for diabetic complications, most have focused on the presence or absence of complications rather than their cumulative burden or stage-specific progression. However, diabetic complications are not static events but dynamic processes that evolve across different stages, ranging from no complications to multiple coexisting complications. Understanding how risk factors influence both the spectrum and burden of complications across these stages is crucial for improving risk stratification and clinical management.

Age is a key determinant in the development of diabetic complications, but its role may not be uniform across disease stages (8). In addition, the interaction between age and diabetes duration may further modify the risk of complications, reflecting a complex and dynamic interplay between aging and disease progression. Advanced statistical approaches, such as restricted cubic spline models, interaction analysis, and partial proportional odds models, provide an opportunity to capture these nonlinear, interactive, and stage-dependent relationships.

Therefore, the present study aimed to comprehensively investigate association between age and spectrum and burden of diabetic complications in patients with T2DM. By integrating multiple analytical approaches, we sought to elucidate the dynamic effects of age and its interaction with diabetes duration across different stages of complication burden, thereby providing more refined evidence for clinical risk assessment and management.

## 2 Materials and methods

### 2.1 Inclusion criteria

All medical records were admitted to the Department of Endocrinology of PLA General Hospital with T2DM from January 2019 to December 2023.

The diagnostic criteria all met the diagnostic criteria of diabetes of the World Health Organization and the international Diabetes Federation (9, 10).

### 2.2 Exclusion criteria

Patients with severe systemic disease or cachexia;

Patients not hospitalized for T2DM;

Patients with malignant tumors or severe liver, renal, or cardiac failure;

Patients with incomplete clinical data.

### 2.3 Definitions of diabetic complications and outcome ascertainment

This cross-sectional study included patients with type 2 diabetes mellitus (T2DM) recruited from Department of Endocrinology of PLA General Hospital between 2019 and 2023. Demographic, clinical, and laboratory data were extracted from electronic medical records using standardized procedures.

The primary outcomes were (1) the presence of any diabetic complication (yes/no) and (2) complication burden, defined as the number of complications per patient and categorized as 0, 1, 2, or ≥3. Diabetic complications were classified into macrovascular complications, microvascular complications, and peripheral neuropathy.

Macrovascular complications were defined as the presence of coronary heart disease/chronic coronary disease, cerebrovascular disease, or peripheral arterial disease, based on documented clinical diagnoses, discharge diagnoses, imaging reports, or specialist records.

Microvascular complications included diabetic kidney disease and diabetic retinopathy. Diabetic kidney disease was ascertained based on documented persistent albuminuria/proteinuria and/or reduced estimated glomerular filtration rate, in accordance with guideline-based chronic kidney disease definitions. Diabetic retinopathy was defined according to funduscopic examination findings, retinal imaging reports, or ophthalmologic diagnoses recorded in the medical records.

Peripheral neuropathy was defined based on a documented diagnosis of diabetic peripheral neuropathy and/or compatible neurological symptoms and signs recorded by treating physicians, with neurophysiological examination results used when available.

The main exposure was age (years). Covariates included sex, diabetes duration, body mass index, systolic blood pressure, HbA1c, creatinine, triglycerides, HDL-C, and LDL-C. Missing data were minimal (< 5%). Potential confounders were selected based on clinical relevance.

### 2.4 Data extraction and quality control

Data were extracted from the electronic medical record system, laboratory information system, and specialist records using a standardized case report form. Demographic characteristics, clinical history, blood pressure, anthropometric measurements, and laboratory variables were collected.

Age was calculated at the index date. Diabetes duration was defined as the interval between the first documented diagnosis of type 2 diabetes and the index date. For laboratory variables, the measurement closest to the index date was used. When multiple measurements were available within the same admission, the first available result was selected according to a predefined protocol.

Two investigators independently extracted and cross-checked all key variables. Discrepancies were resolved by discussion and, when necessary, adjudication by a senior investigator. This standardized extraction process was used to improve reproducibility and reduce information bias.

### 2.5 Statistics analysis

All statistical analyses were performed using R software (version 4.3.3). Before model fitting, the extent of missingness was summarized for all study variables, and the overall missing-data pattern was assessed. Because the proportion of missing data was low for all analytical variables (<5%), missing values in the primary analyses were handled using multiple imputation by chained equations (MICE) to reduce potential bias and improve statistical efficiency. Within the MICE framework, continuous variables with missing values were imputed using predictive mean matching. The imputation model included the outcome, exposure, covariates, and relevant auxiliary variables. Parameter estimates from the imputed datasets were combined using Rubin’s rules.

Continuous variables are presented as mean ± standard deviation or median (interquartile range), as appropriate, and categorical variables are presented as counts and percentages. Group comparisons were performed using analysis of variance, the Kruskal–Wallis test, or the chi-square test, as appropriate, according to variable distribution and type.

Binary logistic regression was used to evaluate the association between age and the presence of diabetic complications, and multivariable models were fitted in a stepwise manner. Ordinal logistic regression was used to assess the association between age and complication burden categorized as 0, 1, 2, and ≥3. When the proportional odds assumption was violated, a partial proportional odds (PPO) model was applied.

Restricted cubic spline (RCS) analysis was performed to explore the potential nonlinear association between age and diabetic complications. The final number of knots was determined by comparing model fit across candidate spline models while also considering interpretability. Interaction terms were further introduced to assess whether diabetes duration modified the association between age and diabetic complications, and interaction effects were evaluated using likelihood ratio tests.

Multicollinearity among candidate covariates was assessed using variance inflation factors (VIFs). As sensitivity analyses, all primary models were refitted using complete-case data and compared with the multiply imputed results. In addition, baseline characteristics were compared between participants with complete data and those with any missing values to evaluate the potential impact of missingness on the study findings. All tests were two-sided, and P < 0.05 was considered statistically significant. For analyses involving multiple comparisons, FDR-adjusted P values were used to determine statistical significance.

## 3 Results

### 3.1 Baseline characteristics of diabetic patients with and without complications

A total of 489 diabetic patients were included, including 119 without complications and 370 with complications. Compared with the non-complication group, patients with complications were older and had a longer disease duration, higher systolic blood pressure, BMI, BUN, Cr, CysC, HCY, and SA levels (all P<0.05). In contrast, HbA1c, GA%, HB, RBC, and SOD levels were significantly lower in the complication group (all P<0.05). No significant differences were observed between the two groups in sex, diastolic blood pressure, height, weight, Glu, UA, TG, HDL, LDL, WBC, NEU, or PLT (all P>0.05).Details are presented in **Table 1**. Further stratification by the number of complications showed that the differences observed between patients with and without complications in **Table 1** generally remained consistent. With an increasing number of complications, age, disease duration, systolic blood pressure, and levels of BUN, Cr, CysC, HCY, and SA showed an overall increasing trend, whereas HB, RBC, and SOD showed an overall decreasing trend; HbA1c and GA% also differed significantly among groups (all P<0.05). The remaining variables were not significantly different across groups. Details are presented in **Supplementary Table 1**.

**Table 1:** Baseline characteristics of diabetic patients by complication status.

Characteristics	Total (n = 489)	Without complications (n = 119)	With complications (n = 370)	*P*
Age, years	54.00 (47.00, 62.00)	46.00 (34.50, 53.00)	57.00 (50.00, 64.00)	<0.001
Gender, [Male,n(%)]	313 (64.01)	79 (66.39)	234 (63.24)	0.534
diabetes duration, years	10.00 (4.00, 15.00)	3.00 (0.80, 10.00)	12.00 (7.00, 16.00)	<0.001
Systolic pressure, mmHg	130.00 (120.00, 146.00)	124.00 (117.50, 132.00)	135.00 (124.00, 150.00)	<0.001
Diastolic pressure, mmHg	80.00 (73.00, 90.00)	80.00 (70.00, 88.00)	80.00 (74.00, 90.00)	0.156
Height, cm	167.00 (160.00, 172.00)	168.00 (160.00, 172.50)	167.00 (160.00, 172.00)	0.702
Weight, kg	73.20 (64.50, 82.50)	73.00 (63.75, 81.10)	74.00 (65.00, 83.00)	0.280
BMI, kg/m^2^	26.40 (24.30, 28.70)	25.40 (23.85, 28.20)	26.70 (24.33, 28.90)	0.022
HbA1c, mmol/L	8.60 (7.40, 10.00)	9.10 (7.90, 10.65)	8.40 (7.20, 9.80)	<0.001
GA, %	21.44 (16.43, 26.94)	23.52 (18.00, 28.61)	20.83 (15.92, 26.46)	0.001
Glu, mmol/L	7.79 (6.07, 10.21)	8.66 (6.47, 11.35)	7.53 (5.93, 10.01)	0.076
BUN, mmol/L	5.26 (4.29, 6.42)	4.66 (4.00, 5.64)	5.42 (4.42, 6.59)	<0.001
Cr, μmol/L	69.10 (58.60, 81.60)	67.40 (59.30, 76.85)	70.20 (58.12, 85.72)	0.04
UA, μmol/L	312.30 (259.30, 376.60)	295.90 (253.70, 348.45)	320.25 (259.77, 385.73)	0.097
TG, mmol/L	1.53 (1.09, 2.24)	1.53 (1.02, 2.38)	1.54 (1.13, 2.22)	0.704
HDL, mmol/L	1.01 (0.85, 1.24)	1.04 (0.85, 1.29)	1.00 (0.86, 1.23)	0.348
LDL, mmol/L	2.77 (2.11, 3.33)	2.89 (2.29, 3.38)	2.71 (2.07, 3.33)	0.062
HB, g/L	141.00 (128.00, 152.00)	144.00 (131.50, 156.50)	139.00 (127.00, 150.00)	0.002
WBC, 10^9/L	6.20 (5.18, 7.54)	6.29 (5.24, 7.62)	6.20 (5.19, 7.53)	0.515
RBC, 10^12/L	4.63 (4.26, 5.02)	4.77 (4.47, 5.21)	4.59 (4.21, 4.90)	<0.001
NEU	0.56 (0.51, 0.62)	0.55 (0.50, 0.61)	0.57 (0.51, 0.63)	0.252
PLT, 10^9/L	211.00 (173.00, 252.00)	213.00 (174.00, 256.00)	208.00 (173.00, 246.75)	0.273
CysC, mg/L	0.87 (0.76, 1.04)	0.80 (0.69, 0.90)	0.90 (0.79, 1.09)	<0.001
HCY, μmol/L	10.80 (8.30, 13.60)	9.60 (7.35, 12.55)	11.35 (8.80, 13.90)	<0.001
SA, mg/dL	56.70 (51.10, 62.90)	54.60 (50.25, 61.10)	57.30 (51.62, 63.30)	0.045
SOD, U/mL	144.70 (128.40, 162.50)	151.00 (132.90, 169.05)	142.95 (127.32, 160.12)	0.014

### 3.2 Distribution of diabetic complications across age groups

The distribution of diabetic complications across age groups is presented in **Table 2**.

**Table 2:** Distribution of diabetic complications across age groups.

Characteristics	Total (n = 489)	<45 years (n = 107)	45–54 years (n = 138)	55–64 years (n = 145)	≥65 years (n = 99)	*P*
Complication status, n (%)	370 (75.66)	49 (45.79)	103 (74.64)	127 (87.59)	91 (91.92)	<0.001
Microvascular complications, n (%)						
MaVD	141 (28.83)	17 (15.89)	35 (25.36)	52 (35.86)	37 (37.37)	<0.001
PN	149 (30.47)	13 (12.15)	33 (23.91)	57 (39.31)	46 (46.46)	<0.001
MVD	325 (66.46)	37 (34.58)	90 (65.22)	114 (78.62)	84 (84.85)	<0.001

MaVD, macrovascular complications; PN, peripheral neuropathy; MVD, microvascular disease.

Data are presented as number (percentage). P values were calculated using the chi-square test.

Overall, the prevalence of any diabetic complication increased significantly with age (P < 0.001). The proportion of patients with complications rose from 45.79% in individuals aged < 45 years to 91.92% in those aged ≥ 65 years, indicating a strong age-related increase in complication risk. A similar trend was observed for macrovascular complications, with prevalence increasing progressively from 15.89% in the youngest group to 37.37% in the oldest group (P < 0.001). Peripheral neuropathy also showed a marked age-related increase, rising from 12.15% in patients aged < 45 years to 46.46% in those aged ≥ 65 years (P < 0.001). Microvascular disease exhibited the most pronounced increase across age groups, with prevalence rising from 34.58% in the youngest group to 84.85% in the oldest group (P < 0.001).

Taken together, these findings demonstrate a clear and consistent age-dependent increase in both the prevalence and burden of diabetic complications.

### 3.3 Univariate logistic regression analysis of factors associated with diabetic complications

Univariate logistic regression analysis showed that age, disease duration, systolic blood pressure, BMI, BUN, Cr, CysC, and HCY were positively associated with the risk of diabetic complications (all P<0.05). In contrast, HbA1c, GA%, HDL, LDL, HB, and RBC were negatively associated with the risk of diabetic complications (all P<0.05). These findings were generally consistent with the distribution pattern in **Table 1**, where patients with complications had higher age, longer disease duration, higher systolic blood pressure, renal function-related indicators, and HCY levels, but lower HbA1c, GA%, HB, and RBC levels. The remaining variables were not significantly associated with diabetic complications. Details are shown in **Table 3**.

**Table 3:** Univariate logistic regression analysis of factors associated with diabetic complications.

Characteristics	OR (95%CI)	*P*
Age, years	1.09 (1.06,1.11)	<0.001
Gender		
Male	1.00 (Reference)	
Femal	1.15 (0.74,1.77)	0.534
diabetes duration, years	1.16 (1.12,1.21)	<0.001
Systolic pressure, mmHg	1.04 (1.03,1.06)	<0.001
Diastolic pressure, mmHg	1.01 (0.99,1.03)	0.166
BMI, kg/m^2^	1.06 (1.01,1.13)	0.045
HbA1c, mmol/L	0.82 (0.74,0.91)	<0.001
GA, %	0.96 (0.94,0.99)	0.004
Glu, mmol/L	0.96 (0.91,1.02)	0.196
BUN, mmol/L	1.42 (1.22,1.66)	<0.001
Cr, μmol/L	1.01 (1.01,1.03)	0.009
UA, μmol/L	1.00 (1.00,1.00)	0.363
TG, mmol/L	0.96 (0.87,1.05)	0.326
HDL, mmol/L	0.53 (0.32,0.91)	0.020
LDL, mmol/L	0.78 (0.64,0.95)	0.013
HB, g/L	0.98 (0.97,0.99)	0.001
WBC, 10^9/L	0.97 (0.87,1.08)	0.607
RBC, 10^12/L	0.43 (0.28,0.64)	<0.001
NEU	0.92 (0.63,1.35)	0.663
PLT, 10^9/L	1.00 (0.99,1.00)	0.337
CysC, mg/L	22.92 (6.80,77.21)	<0.001
HCY, μmol/L	1.07 (1.02,1.12)	0.003
SA, mg/dL	1.02 (1.00,1.04)	0.071
SOD, U/mL	1.00 (1.00,1.00)	0.670

### 3.4 Independent association between age and diabetic complications

Before conducting multivariable logistic regression analyses, correlation and collinearity diagnostics were performed for all candidate variables. **Supplementary Figure 1** showed relatively strong correlations among glycemic markers, including HbA1c, GA, and Glu, as well as among renal function indicators, including BUN, creatinine, and CysC. **Supplementary Figure 2** further demonstrated that most variables had variance inflation factors (VIFs) below 5, whereas CysC showed a relatively higher VIF. Considering pairwise correlations, collinearity, and clinical relevance, HbA1c was selected as the representative glycemic marker for subsequent multivariable analyses, and CysC was not included in the final models.

As shown in **Table 4**, age was significantly associated with diabetic complications in all three binary logistic regression models (all P<0.001). In the fully adjusted model, each one-year increase in age was associated with a 6% higher risk of having diabetic complications (OR = 1.06, 95% CI: 1.03–1.08), indicating that age was an independent correlate of diabetic complications.

**Table 4:** Association between age and diabetic complications across different models.

Characteristics	Model1 OR (95%CI)	*P*	Model2 OR (95%CI)	*P*	Model3 OR (95%CI)	*P*
Age(years)	1.09 (1.06, 1.11)	<0.001	1.06 (1.04,1.08)	<0.001	1.06 (1.03, 1.08)	<0.001

Model 1: Crude model.

Model 2: Adjusted for sex, diabetes duration, systolic blood pressure, and body mass index.

Model 3: Further adjusted for sex, diabetes duration, systolic blood pressure, body mass index, HbA1c, creatinine, triglycerides, HDL-C, and LDL-C.

On this basis, partial proportional odds (PPO) models were further applied to examine the association between age and complication burden across ordered complication groups. It should be noted that the odds ratios derived from PPO models are estimated at different cumulative thresholds, and their direction and magnitude depend on the coding of the ordinal outcome and model parameterization. Therefore, they should not be directly compared numerically with the odds ratios from the binary logistic regression model. Importantly, the overall interpretation was consistent across the two approaches, namely that age was closely associated with diabetic complications.

The PPO analysis further showed that this association was stage-dependent. In the crude model, age was significantly associated with complication burden across all cumulative thresholds (all P<0.001). After adjustment, age remained significantly associated with the transition from no complications to any complications and from fewer to more complications, whereas its association with the highest level of complication burden (0/1/2 vs 3) was no longer significant. These findings suggest that age mainly contributes to the occurrence and early accumulation of diabetic complications, while its influence becomes less pronounced at more advanced stages of complication burden.

In addition, diabetes duration and systolic blood pressure were consistently associated with greater complication burden, whereas the associations of sex and BMI were weaker or inconsistent. HbA1c and lipid-related variables were not significantly associated with complication burden in the fully adjusted model. Detailed results are presented in **Supplementary Table 2**.

### 3.5 Association between age and diabetic complication subtypes

Given the association of age with overall diabetic complications and number of complications, its associations with specific complication subtypes were further evaluated. In the crude model, age was significantly associated with MaVD, PN, and MVD (all P<0.001). After partial adjustment, age remained significantly associated with PN and MVD, whereas the association with MaVD was no longer significant. In the fully adjusted model, age remained significantly associated with MVD (OR = 1.06, 95% CI: 1.04–1.09, P<0.001), while the association with PN was attenuated to borderline significance (OR = 1.02, 95% CI: 1.00–1.04, P = 0.051), and no significant association was observed for MaVD (P = 0.75). These findings suggest that the effect of age differs across complication subtypes and is more robust for microvascular disease. Details are shown in **Table 5**.

**Table 5:** Association between age and diabetic complication subtypes in logistic regression models.

Complication subtype	Model 1 OR (95% CI)	*P*	Model 2 OR (95% CI)	*P*	Model 3 OR (95% CI)	*P*
MaVD	1.03 (1.02,1.05)	<0.001	1.00 (0.98,1.02)	0.980	1.00 (0.98,1.02)	0.750
PN	1.05 (1.04,1.07)	<0.001	1.02 (1.01,1.04)	0.028	1.02 (1.00,1.04)	0.051
MVD	1.08 (1.06,1.10)	<0.001	1.07 (1.04,1.09)	<0.001	1.06 (1.04,1.09)	<0.001

MaVD, macrovascular complications; PN, peripheral neuropathy; MVD, microvascular disease; OR, odds ratio; CI, confidence interval.

Model 1: Crude model.

Model 2: Adjusted for sex, diabetes duration, systolic blood pressure, and body mass index.

Model 3: Further adjusted for sex, diabetes duration, systolic blood pressure, body mass index, HbA1c, creatinine, triglycerides, HDL-C, and LDL-C.

### 3.6 Interaction between age and diabetes duration

**Figure 1:**
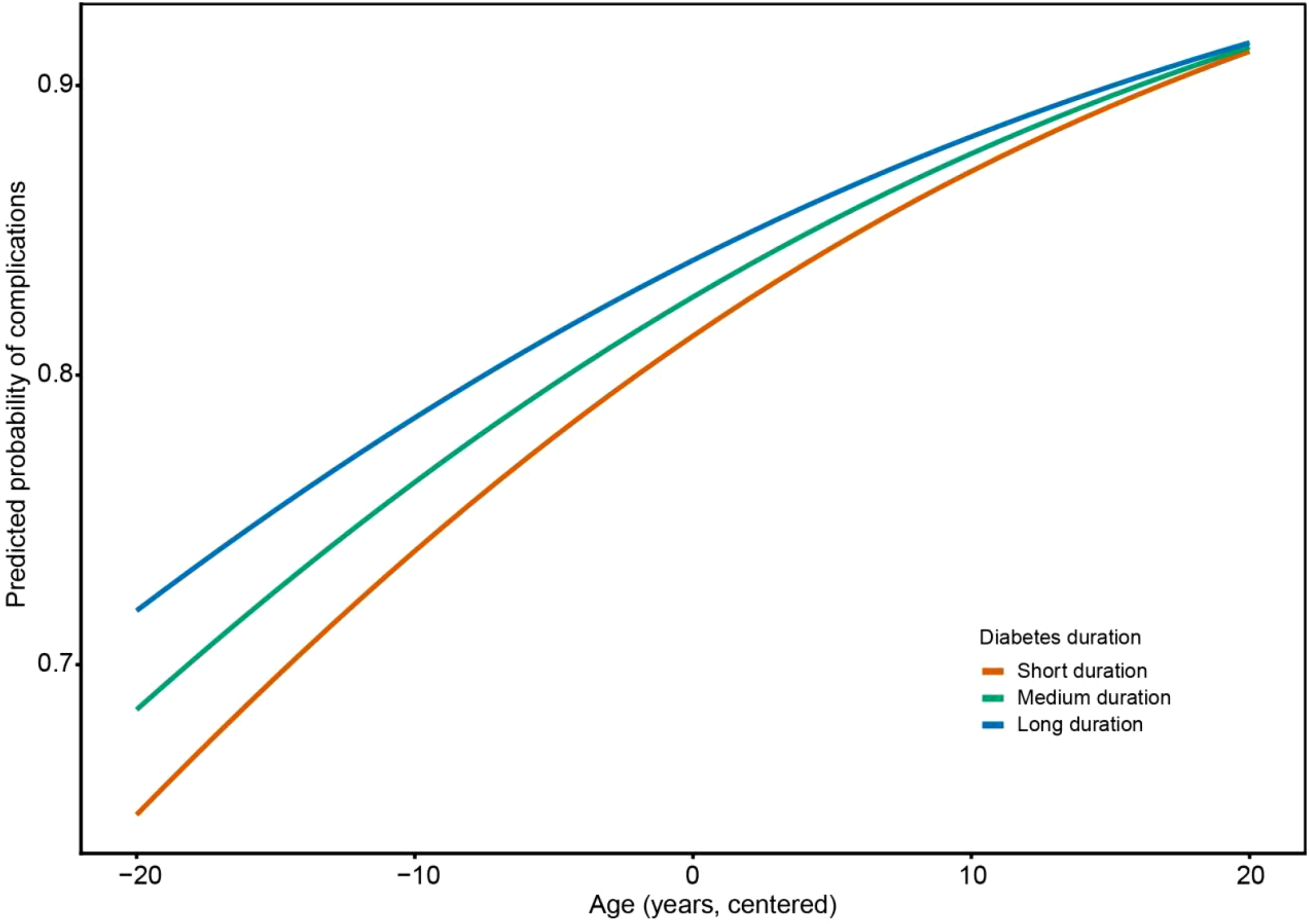
Interaction between age and diabetes duration on the predicted probability of diabetic complications in the fully adjusted model. Predicted probabilities were derived from the fully adjusted logistic regression model (Model 3). Age was centered, and diabetes duration was categorized into short, medium, and long duration groups. Other covariates were held constant at reference or mean values.

**Figure 1** shows an interaction between age and diabetes duration on the risk of diabetic complications in the fully adjusted model. Overall, the predicted probability of complications increased with age across all duration groups, and patients with longer diabetes duration consistently had a higher predicted risk than those with shorter duration. Meanwhile, the effect of age differed by disease duration: the increase in predicted risk with age was steeper in the short-duration group but more gradual in the long-duration group, suggesting that the effect of age on complication risk attenuated as diabetes duration increased. **Supplementary Table 3** further confirmed that, in the fully adjusted model, both age (OR = 1.040, 95% CI: 1.014–1.068, P = 0.003) and diabetes duration (OR = 1.096, 95% CI: 1.049–1.147, P<0.001) were significantly associated with diabetic complications. A significant interaction between age and diabetes duration was also observed (OR = 0.996, 95% CI: 0.993–1.000, P = 0.023). Because the interaction term was less than 1, the association between age and complication risk appeared to weaken as diabetes duration increased. In addition, BMI and systolic blood pressure were positively associated with complication risk, whereas sex, HbA1c, creatinine, and lipid-related variables were not significantly associated in this model. These findings suggest that age plays a stronger role in the early stages of disease, whereas diabetes duration becomes the dominant factor as the disease progresses.

### 3.7 Interaction between age and diabetes duration on the number of complications

**Figure 2:**
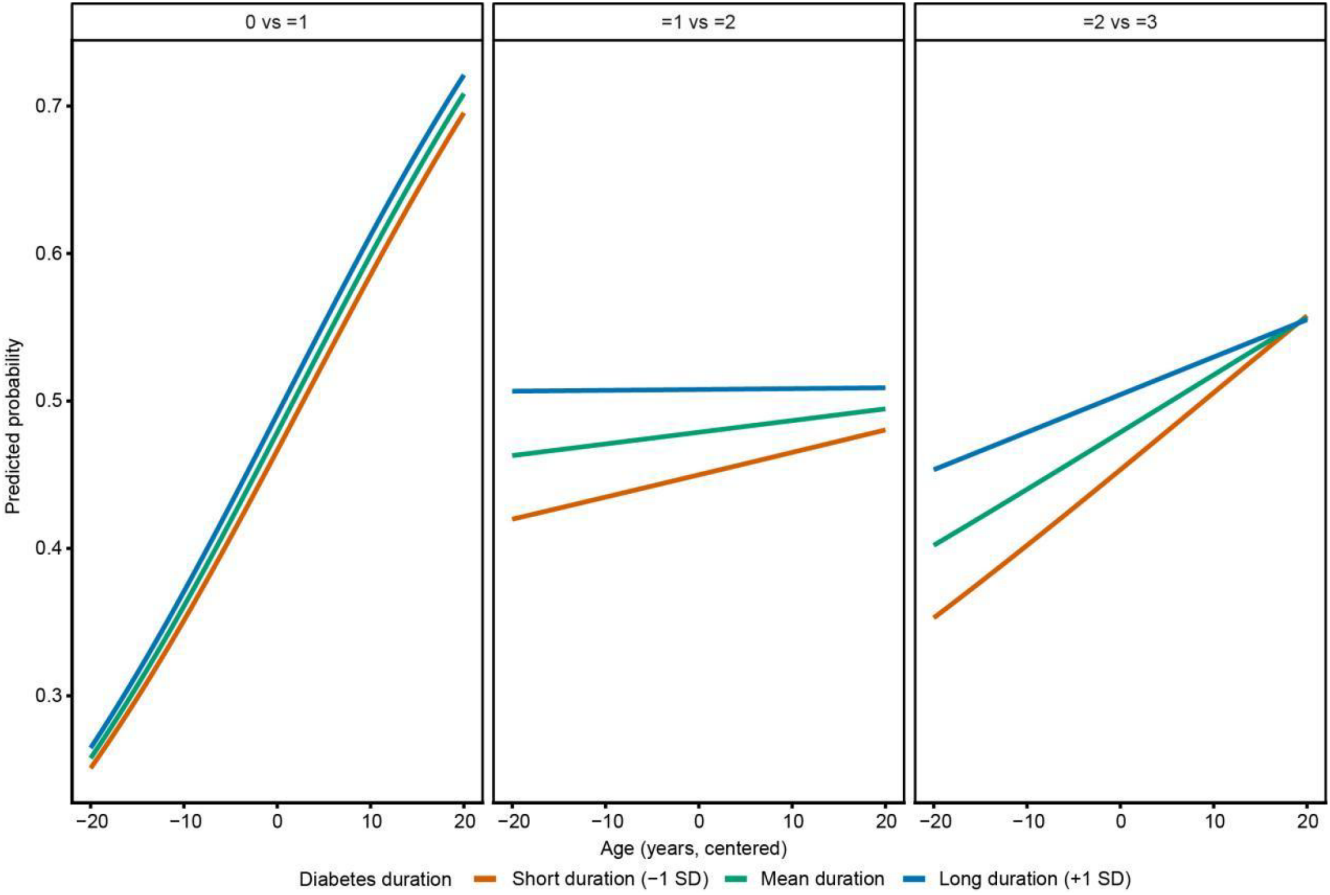
Stage-specific interaction between age and diabetes duration on number of complications based on partial proportional odds models. 0, 0 complication group; 1, 1 complication group; 2, 2 complications group; 3, 3: complications group.

**Figure 2** shows that the effects of age and diabetes duration on the number of complications differed across stages. Overall, the predicted probability of being in a higher complication-count category increased with age in all duration groups, but the patterns were not the same. At the transition from no complications to at least one complication, the increase with age was more obvious in patients with shorter diabetes duration. As the number of complications increased, the differences between duration groups diminished. At the highest complication-count threshold, patients with longer diabetes duration had higher overall predicted probabilities, and the age-related changes were more similar across groups. Taken together, these results suggest an interaction between age and diabetes duration, and this interaction changes as the number of complications increases.The results of **Supplementary Table 4** were generally consistent with those of **Figure 2**. After full adjustment, the effect of age was mainly observed in the transition from a lower to a moderate number of complications. Meanwhile, diabetes duration and systolic blood pressure remained associated with a higher number of complications, whereas HbA1c, creatinine, and lipid-related variables were not significantly associated after full adjustment. Detailed results are shown in **Supplementary Table 4**.

### 3.8 Restricted cubic spline analysis of the association between age and diabetic complications

**Figure 3:**
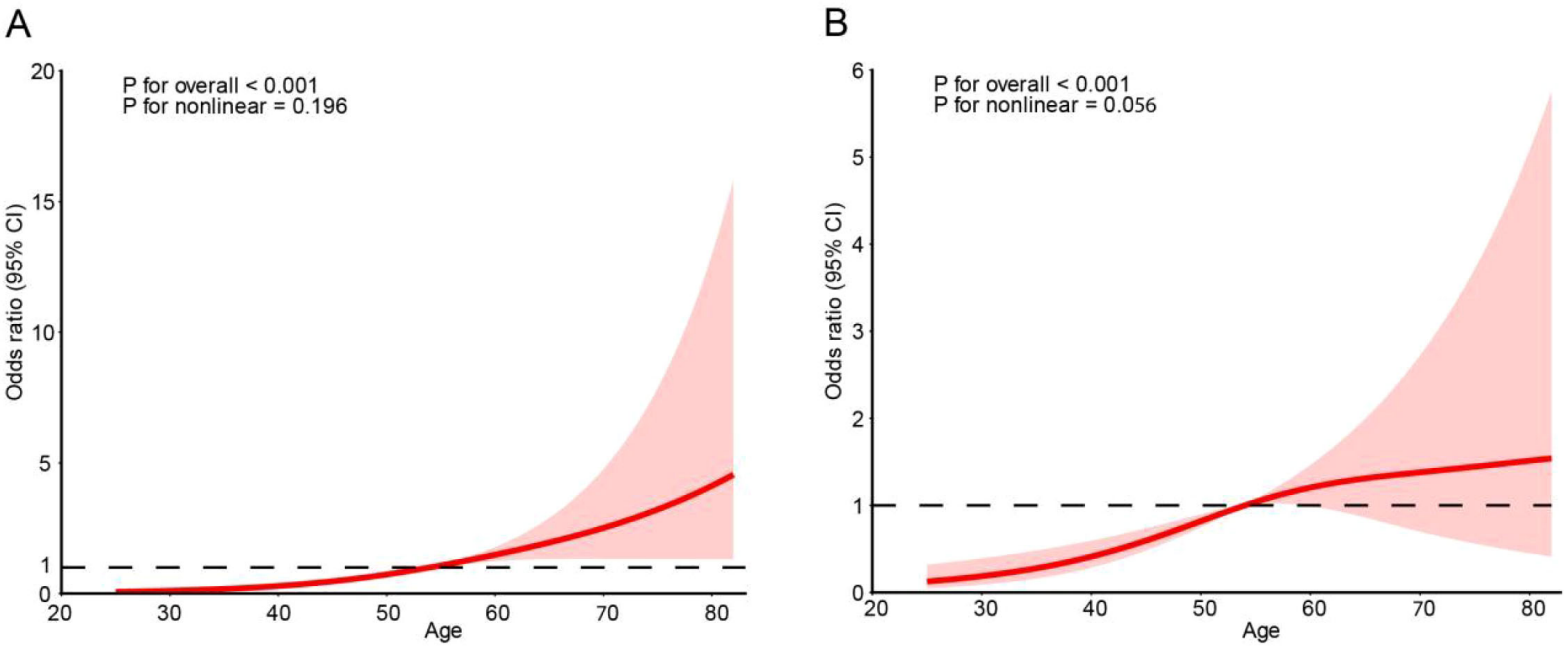
Restricted cubic spline analysis of the association between age and diabetic complications in crude and fully adjusted models. The solid red line represents the estimated odds ratios, and the shaded area indicates 95% confidence intervals. The dashed horizontal line represents an odds ratio of 1. Panel **(A)** shows the crude model (Model 1), and Panel **(B)** shows the fully adjusted model (Model 3). The models were adjusted for sex, diabetes duration, systolic blood pressure, body mass index, HbA1c, creatinine, triglycerides, HDL-C, and LDL-C.

**Supplementary Table 5** shows that among the restricted cubic spline models with different numbers of knots, the model with 3 knots had the lowest AIC and BIC values (428.19 and 491.08, respectively), indicating the best balance between model fit and complexity. As the number of knots increased, both AIC and BIC gradually increased, suggesting that additional knots did not improve model performance. Therefore, the spline analyses were performed using 3 knots. Detailed results are shown in **Supplementary Table 5**. **Figure 3** shows that age was positively associated with the risk of diabetic complications in both the crude and fully adjusted models (overall P < 0.001 for both). In the crude model, the risk of complications increased with age, with a steeper upward trend at older ages, although the confidence intervals became wider in the upper age range. In the fully adjusted model, this association remained significant, with a smoother overall trend and no clear evidence of nonlinearity (P for nonlinear = 0.056). Overall, age was associated with an increased risk of diabetic complications, and this association persisted after adjustment, with an overall largely linear pattern.

### 3.9 Sensitivity analyses and missing-data assessment

Missing data were minimal across all analytical variables, with all missing proportions below 5% (**Supplementary Table 6**). Baseline characteristics were broadly comparable between participants with complete data and those with any missing values (**Supplementary Table 7**). In sensitivity analyses, the complete-case results were generally consistent with those from the multiply imputed analyses in both direction and magnitude of effect estimates (**Supplementary Tables 8**, **S9**). In addition, the restricted cubic spline curves derived from Model 3 were largely unchanged between the two methods (**Supplementary Figure 3**), lending further support to the robustness of the primary results.

## 4 Discussion

Type 2 diabetes mellitus is a common chronic metabolic disorder in clinical practice. With increasing disease duration, patients progressively develop structural damage in the kidneys, retina, peripheral nerves, and cardiovascular system (11–14). These complications substantially increase the risks of disability and mortality, while also leading to a persistent decline in quality of life and imposing a significant long-term burden on clinical management (13, 15). In the present study, the overall prevalence of diabetic complications reached 75.66%, with a relatively higher proportion of microvascular complications. This finding indicates that complications are highly prevalent in the study population and often involve multiple organ systems rather than being confined to a single organ.

The main findings of this study can be summarized as follows. First, age was independently associated with the presence of diabetic complications, and this association remained robust after adjustment for diabetes duration, systolic blood pressure, body mass index, and metabolic parameters. Second, as the number of complications increased, age, diabetes duration, systolic blood pressure, and renal function-related indicators showed a consistent upward trend. Third, the impact of age differed across complication subtypes, with the most stable association observed for microvascular disease. Fourth, a significant interaction between age and diabetes duration was identified, indicating that the effect of age was more pronounced in patients with shorter disease duration, whereas the influence of long-term hyperglycemic exposure became more dominant as duration increased. Finally, restricted cubic spline analysis demonstrated a positive and approximately linear association between age and complication risk, even after multivariable adjustment.

From the baseline characteristics, patients with complications were older, had longer diabetes duration, and exhibited higher systolic blood pressure. They also showed elevated levels of BUN, Cr, CysC, HCY, and SA, along with lower levels of HB, RBC, and SOD. Multivariable analysis further confirmed that age remained significantly associated with complications after controlling for potential confounders, suggesting that age is not merely a demographic variable but is closely linked to the development of diabetic complications.

Diabetes duration also played a critical role in the progression of complications. In this study, longer duration was associated with both a higher prevalence and a greater number of complications. This relationship remained consistent across interaction models and stratified analyses. Chronic hyperglycemia leads to sustained damage to the microcirculation, renal filtration barrier, peripheral nerves, and vascular endothelium, and this damage accumulates over time (16–19). In this context, age and duration represent different but complementary aspects of disease burden: age reflects biological aging and reduced resilience to injury, whereas duration reflects the length of exposure to metabolic abnormalities. Considering both variables together provides a more comprehensive understanding of the progressive nature of diabetic complications.

Subtype analyses further clarified the role of age. After full adjustment, age showed the most stable association with microvascular disease, a borderline association with peripheral neuropathy, and no independent association with macrovascular disease. These differences likely reflect variations in underlying pathophysiology. Microvascular complications are more directly related to long-term metabolic disturbances and microcirculatory dysfunction (20, 21), and therefore tend to accumulate with advancing age. In contrast, macrovascular disease is influenced by multiple factors, including lipid abnormalities (22, 23), baseline cardiovascular status, and medical interventions, which may attenuate the independent effect of age. Consistent with these findings, age-stratified analyses showed that the overall prevalence of complications increased progressively across age groups. A similar pattern was observed across different complication types, with more pronounced increases in microvascular disease and peripheral neuropathy. These results support the notion that complication development follows a cumulative process and is not entirely random, with microvascular damage potentially representing a key early component in this progression.

The interaction between age and diabetes duration further clarifies their respective roles. In patients with shorter disease duration, increasing age is associated with a markedly higher risk of developing complications. However, as duration increases, the influence of age becomes less pronounced, while the cumulative effect of prolonged hyperglycemic exposure emerges as the dominant factor. This pattern is also supported by analyses stratified by the number of complications, where the effect of age is most evident during the transition from no complications to a moderate number of complications. This finding likely reflects differences in the dominant drivers across disease stages. In the early phase of diabetes, older patients may be more susceptible to metabolic injury due to reduced tissue repair capacity and limited microvascular reserve (24, 25), thereby facilitating the initiation of complications. In contrast, in patients with long-standing disease, sustained structural damage caused by chronic hyperglycemia becomes the primary determinant of disease progression, outweighing the contribution of age (26).

In this study, HbA1c and glycated albumin levels were lower in patients with complications and even showed an inverse association in univariate analyses. Although this finding appears counterintuitive, it is not uncommon in cross-sectional studies. Glycemic markers primarily reflect short-term glucose control and may not capture long-term glycemic exposure prior to the development of complications (27). In addition, patients with established complications are more likely to receive intensive treatment and regular follow-up, leading to better short-term glycemic control. Furthermore, reductions in hemoglobin and impaired renal function, may influence the measurement and interpretation of glycemic markers (28, 29). Therefore, in this context, these indicators should be interpreted as measures of current metabolic status rather than long-term risk predictors.

Elevated systolic blood pressure was consistently associated with complications across multiple models. Hypertension is a well-established contributor to diabetic nephropathy, retinopathy, and cardiovascular damage, highlighting that the development of complications is driven by multiple interacting factors (30, 31). Focusing solely on glycemic control while neglecting blood pressure and disease duration may lead to underestimation of overall risk in clinical practice.

The RCS analysis further supported the primary findings. A positive association between age and complication risk was observed in both crude and adjusted models, with an approximately linear pattern after adjustment. This indicates that the effect of age is gradual and cumulative across the lifespan, rather than confined to a specific age threshold.

This study has several limitations. As a cross-sectional retrospective analysis, it identifies associations rather than causality, and prospective longitudinal studies are needed to confirm these findings. In addition, because the study was conducted in a tertiary hospital, selection bias and limited generalizability should be considered. Information bias related to retrospective data extraction and possible misclassification of complications or covariates cannot be completely excluded. Although we adjusted for major covariates, residual confounding may still remain. Furthermore, while missing data were minimal (<5% for all variables), we addressed this issue using multiple imputation and confirmed the robustness of the results in complete-case sensitivity analyses.

## 5 Conclusions

In conclusion, age is independently associated with the presence of chronic complications in patients with type 2 diabetes, and this association is more consistent for microvascular disease. A significant interaction between age and diabetes duration was observed, indicating that the effect of age is more pronounced in earlier stages of the disease, whereas the cumulative impact of long-term metabolic disturbances becomes dominant as duration increases. Systolic blood pressure is also an important contributor to complication risk. These findings suggest that risk assessment should incorporate age, disease duration, and blood pressure, and that stage-specific screening and targeted interventions—particularly for microvascular complications—may help delay the onset and progression of diabetic complications.

## Data Availability

The raw data supporting the conclusions of this article will be made available by the authors, without undue reservation.
