# Supplementary material for: Age and diabetic complications in type 2 diabetes: the role of disease duration and stage-dependent effects
Source: Front Endocrinol (Lausanne). 2026 May 8;17:1782406. doi: 10.3389/fendo.2026.1782406 (PMC13193961; doi:10.3389/fendo.2026.1782406)
Supplement: Supplementary file 1 [file DataSheet1.docx]

Supplementary Table S1. Baseline characteristics of patients with diabetes according to the number of complications.

| Characteristics | Total (n = 489) | 0 complication  (n = 119) | 1 complications (n = 187) | 2 complications (n = 121) | 3 complications (n = 62) | *P* |
| --- | --- | --- | --- | --- | --- | --- |
|  |  |  |  |  |  |  |
| Age, years | 54.00 (47.00, 62.00) | 46.00 (34.50,53.00) | 54.00 (49.00,62.00) | 57.00 (51.00,64.00) | 61.50 (55.50,69.00) | <0.001 |
| Gender, [Male,n(%)] | 313 (64.01) | 79 (66.39) | 126 (67.38) | 77 (63.64) | 31 (50.00) | 0.090 |
| diabetes duration, years | 10.00 (4.00, 15.00) | 3.00 (0.80,10.00) | 9.00 (3.50,13.50) | 14.00 (10.00,18.00) | 16.00 (11.00,20.00) | <0.001 |
| Systolic pressure, mmHg | 130.00 (120.00, 146.00) | 124.00 (117.50,132.00) | 130.00 (120.00,145.00) | 140.00 (126.00,153.00) | 140.00 (130.00,154.00) | <0.001 |
| Diastolic pressure, mmHg | 80.00 (73.00, 90.00) | 80.00 (70.00,88.00) | 80.00 (74.00,90.00) | 80.00 (73.00,90.00) | 80.00 (75.25,90.00) | 0.517 |
| Height, cm | 167.00 (160.00, 172.00) | 168.00 (160.00,172.50) | 168.00 (160.00,173.00) | 166.00 (161.00,172.00) | 165.50 (157.25,171.00) | 0.491 |
| Weight, kg | 73.20 (64.50, 82.50) | 73.00 (63.75,81.10) | 75.00 (64.25,83.75) | 74.00 (66.00,83.00) | 70.50 (62.25,79.00) | 0.250 |
| BMI, kg/m^2^ | 26.40 (24.30, 28.70) | 25.40 (23.85,28.20) | 26.70 (24.26,28.70) | 26.80 (24.80,29.40) | 26.20 (24.33,28.34) | 0.070 |
| HbA1c, mmol/L | 8.60 (7.40, 10.00) | 9.10 (7.90,10.65) | 8.50 (7.25,10.00) | 8.30 (7.00,9.80) | 8.45 (7.40,9.67) | 0.004 |
| GA, % | 21.44 (16.43, 26.94) | 23.52 (18.00,28.61) | 20.82 (16.51,26.45) | 21.01 (15.17,26.67) | 20.73 (14.94,24.22) | 0.012 |
| Glu, mmol/L | 7.79 (6.07, 10.21) | 8.66 (6.47,11.35) | 7.65 (6.10,9.61) | 7.49 (6.00,10.78) | 7.47 (5.62,10.02) | 0.321 |
| BUN, mmol/L | 5.26 (4.29, 6.42) | 4.66 (4.00,5.64) | 4.92 (4.27,6.24) | 5.60 (4.59,7.07) | 5.99 (5.21,7.80) | <0.001 |
| Cr, μmol/L | 69.10 (58.60, 81.60) | 67.40 (59.30,76.85) | 68.10 (57.15,81.10) | 71.10 (59.00,89.40) | 72.55 (59.48,104.88) | 0.007 |
| UA, μmol/L | 312.30 (259.30, 376.60) | 295.90 (253.70,348.45) | 312.50 (253.85,377.00) | 328.40 (269.10,385.80) | 321.20 (262.00,393.62) | 0.197 |
| TG, mmol/L | 1.53 (1.09, 2.24) | 1.53 (1.02,2.38) | 1.50 (1.06,2.15) | 1.56 (1.17,2.34) | 1.62 (1.19,2.24) | 0.650 |
| HDL, mmol/L | 1.01 (0.85, 1.24) | 1.04 (0.85,1.29) | 1.01 (0.84,1.22) | 0.98 (0.84,1.19) | 1.13 (0.92,1.33) | 0.132 |
| LDL, mmol/L | 2.77 (2.11, 3.33) | 2.89 (2.29,3.38) | 2.73 (2.16,3.31) | 2.69 (1.98,3.38) | 2.57 (1.98,3.11) | 0.209 |
| HB, g/L | 141.00 (128.00, 152.00) | 144.00 (131.50,156.50) | 142.00 (129.00,151.50) | 138.00 (128.00,148.00) | 130.00 (120.00,144.00) | <0.001 |
| WBC, 10^9/L | 6.20 (5.18, 7.54) | 6.29 (5.24,7.62) | 6.02 (5.03,7.08) | 6.60 (5.32,7.85) | 6.35 (5.14,7.68) | 0.058 |
| RBC, 10^12/L | 4.63 (4.26, 5.02) | 4.77 (4.47,5.21) | 4.65 (4.31,4.96) | 4.53 (4.23,4.87) | 4.33 (3.83,4.78) | <0.001 |
| NEU | 0.56 (0.51, 0.62) | 0.55 (0.50,0.61) | 0.57 (0.50,0.62) | 0.57 (0.52,0.64) | 0.56 (0.51,0.62) | 0.449 |
| PLT, 10^9/L | 211.00 (173.00, 252.00) | 213.00 (174.00,256.00) | 205.00 (168.00,245.50) | 210.00 (173.00,252.00) | 209.00 (181.00,238.50) | 0.622 |
| CysC, mg/L | 0.87 (0.76, 1.04) | 0.80 (0.69,0.90) | 0.84 (0.77,0.95) | 0.97 (0.82,1.10) | 1.11 (0.86,1.43) | <0.001 |
| HCY, μmol/L | 10.80 (8.30, 13.60) | 9.60 (7.35,12.55) | 11.50 (8.70,13.50) | 11.70 (9.30,14.60) | 10.75 (8.43,14.78) | 0.002 |
| SA, mg/dL | 56.70 (51.10, 62.90) | 54.60 (50.25,61.10) | 55.60 (51.00,60.75) | 58.40 (52.20,64.60) | 61.25 (52.85,65.68) | 0.002 |
| SOD, U/mL | 144.70 (128.40, 162.50) | 151.00 (132.90,169.05) | 146.90 (129.80,163.60) | 141.70 (124.90,156.40) | 136.70 (118.30,150.17) | 0.001 |

Supplementary Table S2. Partial proportional odds models for the association between age and diabetic number of complications (0–3 groups).

| Characteristics | Model 1 OR (95% CI) | P | Model 2 OR (95% CI) | P | Model 3 OR (95% CI) | P |
| --- | --- | --- | --- | --- | --- | --- |
| Age (0 vs 1/2/3) | 0.926 (0.909, 0.943) | <0.001 | 0.937 (0.922, 0.954) | <0.001 | 0.939 (0.922, 0.955) | <0.001 |
| Age (0/1 vs 2/3) | 0.949 (0.934, 0.964) | <0.001 | 0.978 (0.962, 0.994) | 0.006 | 0.979 (0.964, 0.996) | 0.013 |
| Age (0/1/2 vs 3) | 0.933 (0.912, 0.954) | <0.001 | 1.007 (0.990, 1.023) | 0.425 | 1.009 (0.992, 1.026) | 0.317 |
| Gender |  |  | 0.770 (0.536, 1.106) | 0.158 | 0.661 (0.450, 0.969) | 0.034 |
| Diabetes duration |  |  | 0.900 (0.875, 0.925) | <0.001 | 0.903 (0.878, 0.929) | <0.001 |
| BMI |  |  | 0.944 (0.900, 0.991) | 0.019 | 0.956 (0.909, 1.006) | 0.083 |
| SBP |  |  | 0.979 (0.970, 0.989) | <0.001 | 0.980 (0.970, 0.990) | <0.001 |
| HbA1c |  |  |  |  | 1.045 (0.952, 1.148) | 0.356 |
| Creatinine |  |  |  |  | 0.997 (0.993, 1.000) | 0.062 |
| Triglycerides |  |  |  |  | 0.958 (0.876, 1.048) | 0.353 |
| HDL-C |  |  |  |  | 1.402 (0.789, 2.492) | 0.249 |
| LDL-C |  |  |  |  | 1.079 (0.918, 1.269) | 0.354 |

Abbreviations: 0, 0 complication group; 1, 1 complication group; 2, 2 complications group; 3, 3:complications group; OR, odds ratio; CI, confidence interval; BMI, body mass index; SBP, systolic blood pressure; HDL-C, high-density lipoprotein cholesterol; LDL-C, low-density lipoprotein cholesterol.

Model 1: Crude model.

Model 2: Adjusted for sex, diabetes duration, body mass index, and systolic blood pressure.

Model 3: Further adjusted for sex, diabetes duration, body mass index, systolic blood pressure, HbA1c, creatinine, triglycerides, HDL-C, and LDL-C.

Supplementary Table S3. Multivariable logistic regression analysis of the interaction between age and diabetes duration in relation to diabetic complications.

| Characteristics | Adjusted OR (95% CI) | P value |
| --- | --- | --- |
| Age | 1.040 (1.014, 1.068) | 0.003 |
| Diabetes duration | 1.096 (1.049, 1.147) | <0.001 |
| Age × diabetes duration | 0.996 (0.993, 1.000) | 0.023 |
| Sex | 1.222 (0.684, 2.215) | 0.502 |
| BMI | 1.085 (1.009, 1.168) | 0.029 |
| SBP | 1.030 (1.014, 1.048) | <0.001 |
| HbA1c | 0.933 (0.820, 1.062) | 0.290 |
| Creatinine | 1.005 (0.996, 1.019) | 0.460 |
| Triglycerides | 0.999 (0.872, 1.122) | 0.991 |
| HDL-C | 0.604 (0.262, 1.264) | 0.214 |
| LDL-C | 0.820 (0.648, 1.017) | 0.079 |

Supplementary Table S4. Partial proportional odds models examining the interaction between age and diabetes duration across levels of diabetic number of complications.

| Characteristics | Model 1 OR (95% CI) | P | Model 2 OR (95% CI) | P | Model 3 OR (95% CI) | P |
| --- | --- | --- | --- | --- | --- | --- |
| Age (0 vs >=1) | 0.926 (0.909, 0.943) | <0.001 | 0.937 (0.922, 0.954) | <0.001 | 0.939 (0.922, 0.955) | <0.001 |
| Age (<=1 vs >=2) | 0.949 (0.934, 0.964) | <0.001 | 0.978 (0.962, 0.994) | 0.006 | 0.979 (0.964, 0.996) | 0.013 |
| Age (<=2 vs >=3) | 0.933 (0.912, 0.954) | <0.001 | 1.007 (0.990, 1.023) | 0.425 | 1.009 (0.992, 1.026) | 0.317 |
| Gender |  |  | 0.770 (0.536, 1.106) | 0.158 | 0.661 (0.450, 0.969) | 0.034 |
| Diabetes duration |  |  | 0.900 (0.875, 0.925) | <0.001 | 0.903 (0.878, 0.929) | <0.001 |
| BMI |  |  | 0.944 (0.900, 0.991) | 0.019 | 0.956 (0.909, 1.006) | 0.083 |
| SBP |  |  | 0.979 (0.970, 0.989) | <0.001 | 0.980 (0.970, 0.990) | <0.001 |
| HbA1c |  |  |  |  | 1.045 (0.952, 1.148) | 0.356 |
| Creatinine |  |  |  |  | 0.997 (0.993, 1.000) | 0.062 |
| Triglycerides |  |  |  |  | 0.958 (0.876, 1.048) | 0.353 |
| HDL-C |  |  |  |  | 1.402 (0.789, 2.492) | 0.249 |
| LDL-C |  |  |  |  | 1.079 (0.918, 1.269) | 0.354 |

Abbreviations: 0, 0 complication group; 1, 1 complication group; 2, 2 complications group; 3, 3:complications group; OR, odds ratio; CI, confidence interval; BMI, body mass index; SBP, systolic blood pressure; HDL-C, high-density lipoprotein cholesterol; LDL-C, low-density lipoprotein cholesterol.

Supplementary Table S5. Selection of knot number for restricted cubic spline models based on model fit statistics.

| Number of knots | AIC | BIC | Selected |
| --- | --- | --- | --- |
| 3 | 428.19 | 491.08 | Yes |
| 4 | 430.13 | 497.21 | No |
| 5 | 432.04 | 503.31 | No |
| 6 | 433.3 | 508.76 | No |
| 7 | 435.12 | 514.78 | No |

Abbreviations: AIC, Akaike information criterion; BIC, Bayesian information criterion. Lower AIC and BIC values indicate better model fit with appropriate model complexity.

Supplementary Table S6. Missingness of study variables and imputation methods

| **Variable** | **Missing,n** | **Missing,%** | **Variable type** | **Imputation method** |
| --- | --- | --- | --- | --- |
| SOD, U/mL | 11 | 2.25 | Continuous | Predictive mean matching |
| Height, cm | 7 | 1.43 | Continuous | Predictive mean matching |
| Weight, kg | 7 | 1.43 | Continuous | Predictive mean matching |
| UA, μmol/L | 5 | 1.02 | Continuous | Predictive mean matching |
| HDL, mmol/L | 4 | 0.82 | Continuous | Predictive mean matching |
| LDL, mmol/L | 4 | 0.82 | Continuous | Predictive mean matching |
| TG, mmol/L | 4 | 0.82 | Continuous | Predictive mean matching |
| HB, g/L | 3 | 0.61 | Continuous | Predictive mean matching |
| NEU | 3 | 0.61 | Continuous | Predictive mean matching |
| PLT, 10^9/L | 3 | 0.61 | Continuous | Predictive mean matching |
| RBC, 10^12/L | 3 | 0.61 | Continuous | Predictive mean matching |
| WBC, 10^9/L | 3 | 0.61 | Continuous | Predictive mean matching |
| BUN, mmol/L | 2 | 0.41 | Continuous | Predictive mean matching |
| Cr, μmol/L | 2 | 0.41 | Continuous | Predictive mean matching |
| Glu, mmol/L | 2 | 0.41 | Continuous | Predictive mean matching |
| Diastolic pressure, mmHg | 1 | 0.2 | Continuous | Predictive mean matching |
| Systolic pressure, mmHg | 1 | 0.2 | Continuous | Predictive mean matching |
| HCY, μmol/L | 1 | 0.2 | Continuous | Predictive mean matching |
| SA, mg/dL | 1 | 0.2 | Continuous | Predictive mean matching |
| Age, years | 0 | 0 | Continuous | Fully observed |
| Gender | 0 | 0 | Binary | Fully observed |
| BMI, kg/m2 | 0 | 0 | Continuous | Fully observed |
| diabetes duration, years | 0 | 0 | Continuous | Fully observed |
| HbA1c, mmol/L | 0 | 0 | Continuous | Fully observed |
| GA, % | 0 | 0 | Continuous | Fully observed |
| MaVD | 0 | 0 | Binary | Fully observed |
| MVD | 0 | 0 | Binary | Fully observed |
| PN | 0 | 0 | Binary | Fully observed |

Table note: Overall, the proportion of missing data was low (<5% for all variables).

Supplementary Table S7. Comparison of baseline characteristics between participants with complete data and those with any missing values.

| Characteristics | Complete data | Any missing | *p* |
| --- | --- | --- | --- |
|  | (n = 444) | (n = 45) |  |
| Age, years | 55.00 (47.00, 62.00) | 53.00 (43.00, 63.00) | 0.30 0 |
| Gender, [Male,n(%)] | 284 (64%) | 29 (64%) | >0.900 |
| Diabetes duration, years | 10.00 (4.00, 15.00) | 10.00 (3.00, 16.00) | 0.700 |
| Systolic pressure, mmHg | 130.00 (120.00, 146.00) | 130.00 (120.00, 152.00) | 0.700 |
| Diastolic pressure, mmHg | 80.00 (73.00, 90.00) | 80.00 (77.00, 90.00) | 0.300 |
| Height, cm | 167.00 (160.00, 172.00) | 169.50 (160.00, 173.00) | 0.600 |
| Weight, kg | 73.00 (64.00, 82.00) | 74.50 (66.00, 90.00) | 0.200 |
| BMI, kg/m² | 26.40 (24.25, 28.70) | 27.41 (24.40, 30.00) | 0.200 |
| HbA1c, % | 8.50 (7.40, 9.95) | 9.20 (7.10, 10.40) | 0.400 |
| GA, % | 21.50 (16.46, 27.03) | 19.62 (15.08, 26.54) | 0.500 |
| Glu, mmol/L | 7.63 (5.91, 10.00) | 8.93 (6.22, 12.24) | 0.079 |
| BUN, mmol/L | 5.31 (4.28, 6.36) | 5.01 (4.30, 7.54) | 0.700 |
| Cr, µmol/L | 68.75 (58.75, 81.50) | 69.90 (56.60, 82.10) | >0.900 |
| UA, µmol/L | 312.10 (256.45, 375.10) | 309.35 (268.55, 419.15) | 0.300 |
| TG, mmol/L | 1.53 (1.09, 2.23) | 1.67 (1.11, 5.50) | 0.200 |
| HDL-C, mmol/L | 1.01 (0.86, 1.25) | 0.98 (0.78, 1.18) | 0.054 |
| LDL-C, mmol/L | 2.75 (2.12, 3.36) | 2.86 (1.98, 3.22) | 0.500 |
| HB, g/L | 140.00 (128.00, 151.00) | 142.50 (126.00, 162.00) | 0.600 |
| WBC, ×10⁹/L | 6.21 (5.17, 7.62) | 6.43 (5.50, 7.37) | 0.500 |
| RBC, ×10¹²/L | 4.63 (4.29, 5.01) | 4.66 (4.12, 5.13) | >0.900 |
| NEU, % | 0.56 (0.51, 0.62) | 0.59 (0.51, 0.63) | 0.400 |
| PLT, ×10⁹/L | 211.00 (173.00, 253.00) | 201.50 (165.00, 242.00) | 0.900 |
| CysC, mg/L | 0.88 (0.76, 1.05) | 0.85 (0.77, 0.97) | 0.500 |
| HCY, µmol/L | 10.70 (8.25, 13.60) | 11.50 (9.40, 13.55) | 0.600 |
| SA, mg/dL | 56.60 (51.05, 62.60) | 60.65 (52.60, 65.95) | 0.120 |
| SOD, U/mL | 144.50 (128.40, 161.45) | 145.60 (126.20, 161.50) | 0.700 |

Supplementary Table S8. Comparison of complete-case and multiply imputed results for the primary multivariable logistic regression model

| Variable | Complete-case OR (95% CI) | *P* | Multiply imputed OR (95% CI) | *P* |
| --- | --- | --- | --- | --- |
| Age ,years | 1.055 (1.031, 1.082) | <0.001 | 1.054 (1.030, 1.079) | <0.001 |
| Gender,Female vs Male | 1.319 (0.731, 2.415) | 0.363 | 1.245 (0.697, 2.224) | 0.458 |
| Diabetes duration ,years | 1.091 (1.044, 1.143) | <0.001 | 1.096 (1.047, 1.147) | <0.001 |
| BMI,kg/m² | 1.059 (0.984, 1.140) | 0.125 | 1.076 (1.003, 1.155) | 0.041 |
| Systolic blood pressure,mmHg | 1.030 (1.014, 1.047) | <0.001 | 1.029 (1.013, 1.046) | <0.001 |
| HbA1c ,% | 0.908 (0.797, 1.033) | 0.141 | 0.916 (0.806, 1.041) | 0.179 |
| Cr, μmol/L | 1.004 (0.995, 1.018) | 0.475 | 1.004 (0.993, 1.016) | 0.485 |
| TG, mmol/L | 1.006 (0.885, 1.125) | 0.923 | 0.991 (0.880, 1.115) | 0.875 |
| HDL, mmol/L | 0.563 (0.245, 1.172) | 0.156 | 0.584 (0.268, 1.269) | 0.174 |
| LDL, mmol/L | 0.845 (0.669, 1.047) | 0.135 | 0.844 (0.678, 1.050) | 0.128 |

Odds ratios (ORs) and 95% confidence intervals (CIs) were estimated from the fully adjusted binary logistic regression model. Results from complete-case analysis and multiple imputation were compared to assess robustness.

Supplementary Table S9. Comparison of complete-case and imputed-data analyses for the age × diabetes duration interaction model.

| Characteristics | Complete-case OR (95% CI) | *P* | Imputed-data OR (95% CI) | *P* |
| --- | --- | --- | --- | --- |
| Age ,years | 1.080 (1.045, 1.118) | <0.001 | 1.080 (1.046, 1.117) | <0.001 |
| Diabetes duration,years | 1.316 (1.092, 1.572) | 0.003 | 1.331 (1.109, 1.585) | 0.002 |
| Gender,Female vs Male | 1.289 (0.710, 2.375) | 0.409 | 1.222 (0.684, 2.215) | 0.502 |
| BMI,kg/m² | 1.067 (0.989, 1.151) | 0.093 | 1.085 (1.009, 1.168) | 0.029 |
| Systolic blood pressure,mmHg | 1.031 (1.015, 1.048) | <0.001 | 1.030 (1.014, 1.048) | <0.001 |
| HbA1c,% | 0.921 (0.808, 1.052) | 0.223 | 0.933 (0.820, 1.062) | 0.29 |
| Cr, μmol/L | 1.004 (0.995, 1.018) | 0.477 | 1.005 (0.996, 1.019) | 0.46 |
| TG, mmol/L | 1.009 (0.879, 1.134) | 0.887 | 0.999 (0.872, 1.122) | 0.991 |
| HDL, mmol/L | 0.585 (0.250, 1.250) | 0.199 | 0.604 (0.262, 1.264) | 0.214 |
| LDL, mmol/L | 0.822 (0.647, 1.022) | 0.087 | 0.820 (0.648, 1.017) | 0.079 |
| Age × diabetes duration | 0.997 (0.994, 1.000) | 0.033 | 0.996 (0.993, 1.000) | 0.023 |

Odds ratios (ORs) and 95% confidence intervals (CIs) were estimated from the fully adjusted interaction model. P for interaction by likelihood ratio test: complete-case analysis = 0.042; imputed-data analysis = 0.032.


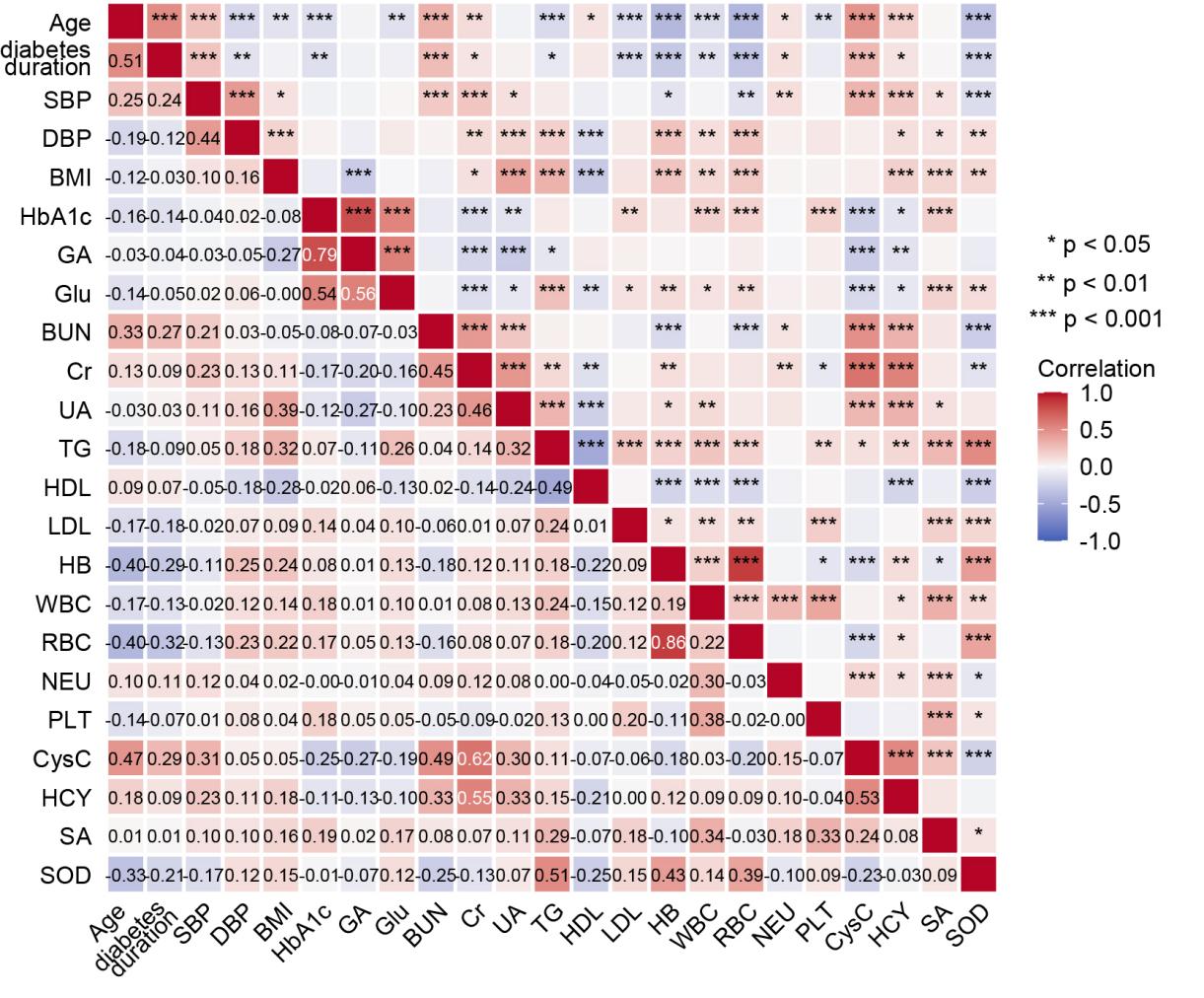


Supplementary Figure S1. Correlation matrix of clinical and biochemical variables in patients with diabetes.

Data are presented as Pearson correlation coefficients. Red indicates positive correlations and blue indicates negative correlations. The intensity of color reflects the strength of the correlation. * P ≤ 0.05; ** P ≤ 0.01; *** P ≤ 0.001.


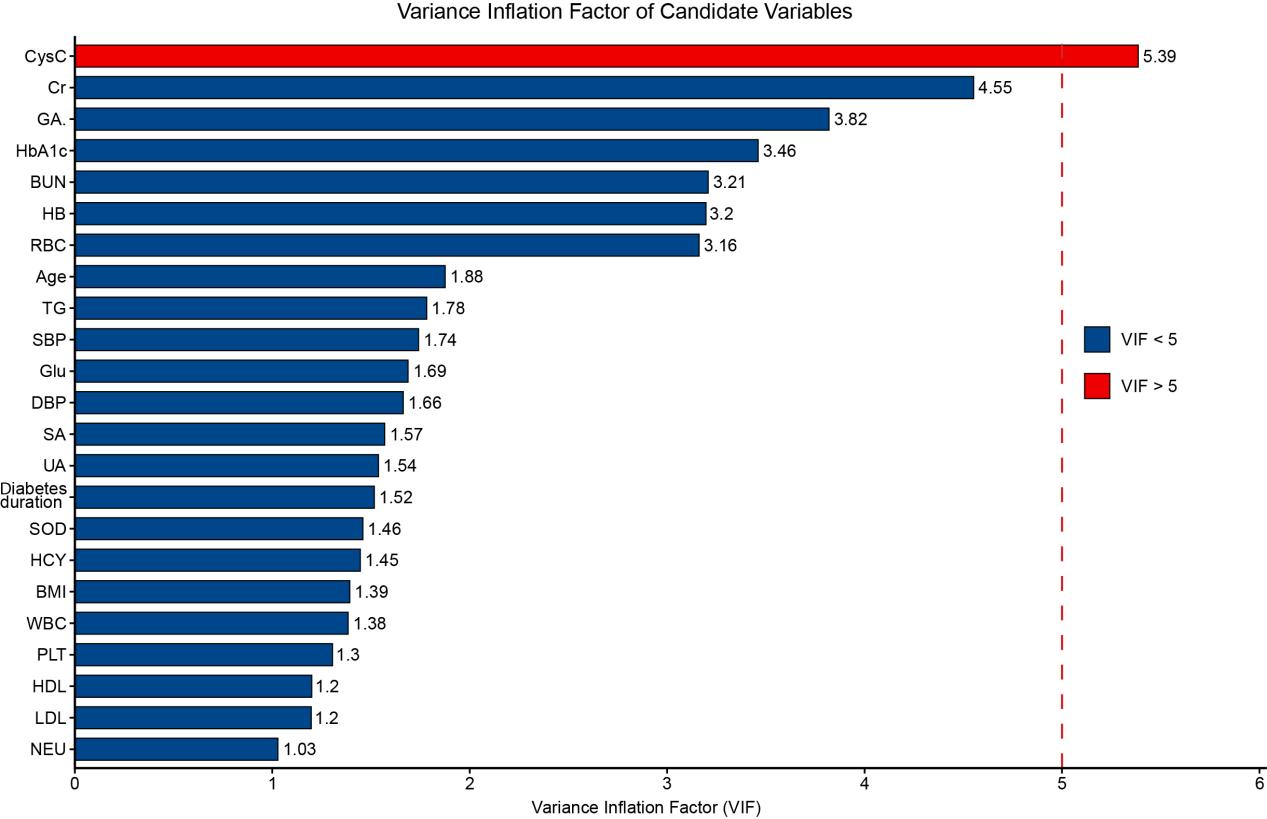
Supplementary Figure S2. Variance inflation factors of candidate variables in the multivariable model.


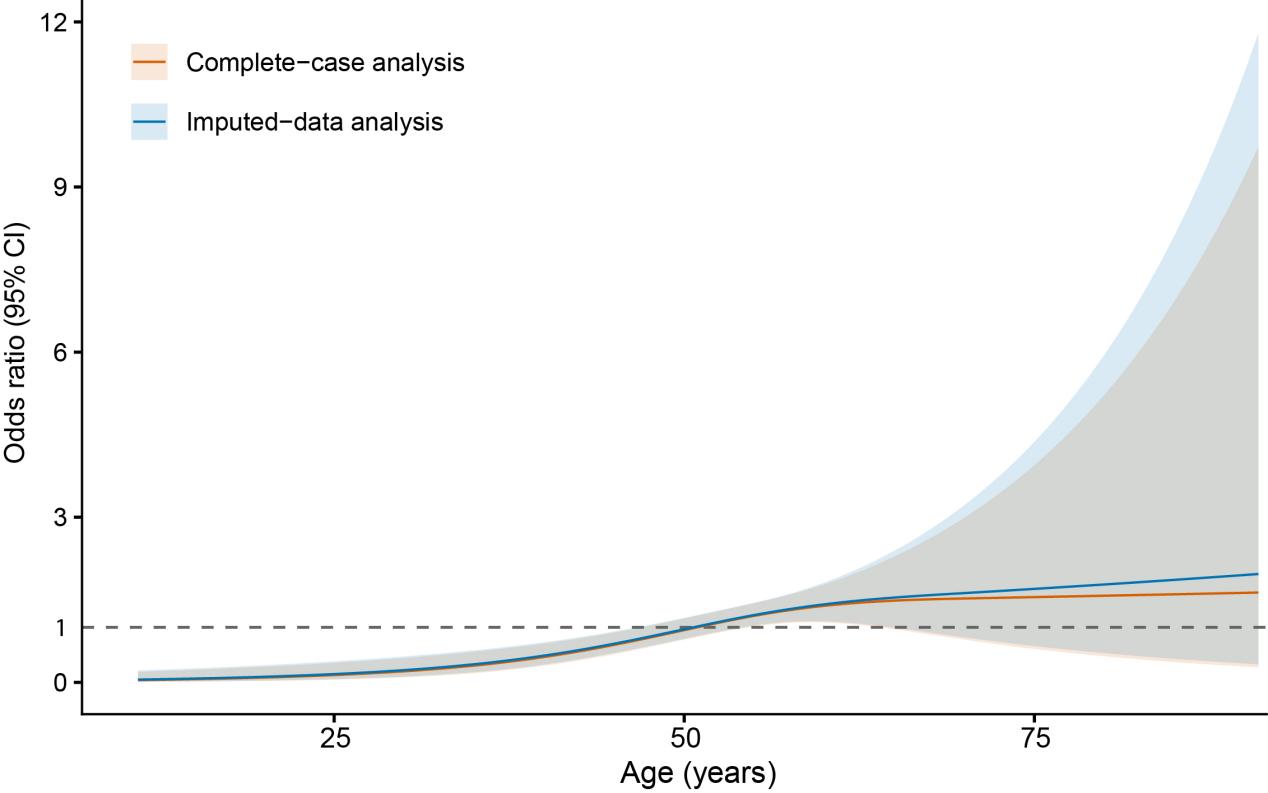


Supplementary Figure S3. Comparison of restricted cubic spline curvesbetween complete-case and imputed-data analyses.

Supplementary Figure S3 shows the fully adjusted model (Model 3). The models were adjusted for sex, diabetes duration, systolic blood pressure, body mass index, HbA1c, creatinine, triglycerides, HDL-C, and LDL-C.
